# Two new species of the bamboo-feeding planthopper genus *Bambusiphaga* Huang & Ding from China (Hemiptera, Fulgoromorpha, Delphacidae)

**DOI:** 10.3897/zookeys.735.21727

**Published:** 2018-02-06

**Authors:** Li Hong-Xing, Lin Yang, Xiang-Sheng Chen

**Affiliations:** 1 Institute of Entomology, Guizhou University, Guiyang,; 2 Guizhou, 550025, P.R. China; 3 The Provincial Special Key Laboratory for Development and Utilization of Insect Resources, Guizhou University, Guiyang, Guizhou, 550025, P.R. China

**Keywords:** Bamboo planthopper, distribution, Fulgoroidea, Homoptera, Oriental region, taxonomy

## Abstract

Two new species of the bamboo-feeding genus *Bambusiphaga* Huang & Ding, 1979, *B. yingjiangensis* **sp. n.** and *B. ventroprocessa* **sp. n.**, are described and illustrated from Yunnan and Hainan, south China. A key to species of the genus are given. A map showing the geographic distribution of the two new species is also provided.

## Introduction

The bamboo-feeding planthopper genus *Bambusiphaga*, belonging to the tribe Tropidocephalini (Delphacidae, Delphacinae), was established by Huang and Ding (1979) (type species: *B. nigropunctata* Huang & Ding, 1979). To date, 25 species are recognized in the genus. Among them, 23 species are distributed in China (Huang et al. 1979; Kuoh et al. 1980; Ding 1982; Ding and Hu 1982; Asche 1983; Ding et al. 1986; Yang and Yang 1986; Qin and Yuan 1999; Chen and Li 2000; Chen et al. 2000; Qin et al. 2006; Chen and Liang 2007; Hou and Chen 2010; Yang and Chen 2011; Qin et al. 2012). Chen and Liang (2007) proposed 20 species of the genus in seven groups: *nigropunctata*, *citricolorata*, *lacticolorata*, *fascia*, *maculata*, *wangmoensis*, and *mirostylis* respectively. Yang and Chen (2011) provided the checklist of 24 species of the genus.

Species of *Bambusiphaga* feed exclusively on bamboo (Bambusoideae). Some of them, such as *B. furca* Huang & Ding, *B. citricolorata* Huang & Tian, *B. taiwanensis* (Muir), *B. lacticolorata* Huang & Ding, *B. maculata* Chen et al. and *B. luodianensis* Ding, are of economic significance since these species have large populations in bamboo fields (Huang et al. 1979; Ding et al. 1986; Yang and Yang 1986; Yang et al. 1999; Chen et al. 2000; Liu and Chen 2008; Zheng and Chen 2013a, b). Specimens have been collected on the leaves of several genera of bamboo, including *Bambusa*, *Dendrocalamus*, *Gelidocalamus*, *Sinocalamus*, *Neosinocalamus*, and *Phyllostachys* (Huang et al. 1979; Ding and Hu 1982; Ding et al. 1986; Yang and Yang 1986; Chen and Li 2000; Chen et al. 2000; Ding 2006; Chen and Liang 2007; Zheng and Chen 2013a, b).

Herein, two new species of *Bambusiphaga*, *B. yingjiangensis* sp. n. and *B. ventroprocessa* sp. n., are described and illustrated from Yunnan and Hainan respectively.

## Materials and methods

Dry male specimens were used for the description and illustration. External morphology was observed under a stereoscopic microscope and characters were measured with an ocular micrometer. Color pictures for adult habitus were obtained by KEYENCE VHX-1000 system. The genital segments of the examined specimens were macerated in 10% KOH and drawn from preparations in glycerin jelly using a Leica MZ 12.5 stereomicroscope. Illustrations were scanned with Canon CanoScan LiDE 200 and imported into Adobe Photoshop 6.0 for labeling and plate composition.

Terminology of morphological and measurements follow Yang and Yang (1986), Chen and Liang (2007), and the morphological terminology of female genitalia follows Bourgoin (1993). Measurements of body length equal the distance between the apex of vertex and tip of tegmen. All measurements are in millimeters (mm).

The type specimens of the new species are deposited in the Institute of Entomology, Guizhou University, Guiyang, China (**IEGU**).

## Taxonomy

### Bambusiphaga

Taxon classificationAnimaliaORDOFAMILIA

Huang & Ding, 1979

Bambusiphaga Huang & Ding, 1979: 170; Asche 1983: 211; Ding and Tian 1983 (in Kuoh et al. 1983): 49; Yang and Yang 1986: 37; Wang and Ding 1996: 22; Ding et al. 1999: 441; Ding 2006: 126; Chen and Liang 2007: 504; Hou and Chen 2010: 392; Yang and Chen 2011: 51.

#### Type species.

*Bambusiphaga nigropunctata* Huang & Ding, 1979, by original designation.

#### Diagnosis.

For the diagnosis and relationships of *Bambusiphaga* see Yang and Chen (2011: 51), Hou and Chen (2010: 392) and Chen and Liang (2007: 504).

#### Host plants.

Bamboo.

#### Distribution.

Oriental region, with highest species diversity in China.

#### Key to species of genus *Bambusiphaga* (male)

(Modified from Yang and Chen 2011 and Qin et al. 2012)

**Table:** 

1	Vertex dark brown or with blackish brown markings	**2**
–	Vertex without any markings	**3**
2	Vertex yellowish brown, basal compartment with a black oval spot in middle part; anal segment without a process, pygofer without medioventral processes (Huang et al. 1979: figs 2, 4)	***B. nigropunctata***
–	Vertex dark brown, basal compartment of vertex without a black oval spot; anal segment with a very long process that surpasses base of genital styles; pygofer with conjugated medioventral processes (Chen and Liang 2007: figs 46, 49)	***B. pianmaensis***
3	Mesonotum with blackish brown markings	**4**
–	Mesonotum without blackish brown markings	**12**
4	Pronotum with blackish brown markings on lateral areas	**5**
–	Pronotum without blackish brown markings on lateral areas	**11**
5	Forewings with basal 1/3 black or with black markings at basal half	**6**
–	Forewings with a large irregular pale brown stripe along transverse vein hence bending along posterior margin to apex (Fig. 8)	***B. yingjiangensis* sp. n.**
6	Forewings with basal 1/3 black	**7**
–	Forewings with large black markings at base	**10**
7	Anal segment without a process on ventral margin (Yang and Chen 2011: fig. 6)	***B. kunmingensis***
–	Anal segment with a very long process on ventral margin	**8**
8	Anal spiny process at left lateroapical angle of anal segment	**9**
–	Anal spiny process at right lateroapical angle of anal segment (Hou and Chen 2010: fig. 14)	***B. basifusca***
9	Pygofer with a medioventral process; aedeagus with two apical processes (Qin et al. 2012: figs 12, 16–17)	***B. taibaishana***
–	Pygofer without medioventral process; aedeagus without apical processes (Ding and Hu 1987: figs 1, 3)	***B. fascia***
10	Forewings with a large black marking at base; anal segment with a long process on ventral margin (Chen et al. 2000: figs 3–4)	***B. maculata***
–	Forewings with two large black markings at base; anal segment without process on ventral margin (Figs 29, 31)	***B. ventroprocessa* sp. n.**
11	Forewings somewhat reddish orange, costal margin blackish brown; genital styles relatively broad and short (Huang et al. 1980: figs 8c, 8f)	***B. nigromariginata***
–	Forewings somewhat yellowish brown, costal margin yellowish brown; genital styles relatively slender (Yang and Yang 1986: figs 20C, 20E; Miur 1917: fig. 44)	***B. taiwanensis***
12	Anal segment with a process on ventral margin	**13**
–	Anal segment without a process on ventral margin	**17**
13	Pygofer with a medioventral process (Muir 1919: fig. 3)	***B. bakeri***
–	Pygofer without a medioventral proces	**14**
14	Anal segment with the process on ventral margin very long, reaching ventral margin of pygofer	**15**
–	Anal segment with the process on ventral margin very short	**16**
15	Genital styles with a process at base, apex rounded (Ding et al. 1986: figs 1 (5–6))	***B. jinghongensis***
–	Genital styles without a process at base, apex forked (Huang et al. 1979: fig. 18)	***B. mirostylis***
16	Tegula with apical 1/2 blackish brown; pygofer with hind margin produced at an acute angle medially; genital styles slender; aedeagus without phallobase (Ding and Hu 1982: figs 1–4)	***B. huangi***
–	Tegula fully yellowish brown; pygofer with hind margin not produced medially; genital styles broad and short; aedeagus with developed phallobase (Chen and Li 2000: figs 11, 13, 15–16)	***B. wangmoensis***
17	Pygofer with a spine on ventral margin	**18**
–	Pygofer without a spine on ventral margin	**20**
18	Genital style with an inversed spine on caudal side near apex which is as long as 1/5 of genital style; aedeagus with three spines subapically (Yang and Chen 2011: figs 20–22)	***B. yangi***
–	Genital style with an angular or tooth-like process on caudal side near apex; aedeagus without spines subapically	**19**
19	Genital styles asymmetrical, right one shorter than left one, without tooth-like process subapically on caudal side; aedeagus with an inversed process on right side near apical 1/3 (Miur 1919: fig. 8)	***B. singaporensis***
–	Genital styles symmetrical; aedeagus without any processes (Ding 1982: figs 3, 5)	***B. luodianensis***
20	Genital styles with a finger-like process at base	**21**
–	Genital styles without a finger-like process at base	**22**
21	Genital styles with a finger-like process subapically; aedeagus curved in middle (Chen and Liang 2007: figs 20–22)	***B. maolanensis***
–	Genital styles with a lamellate process subapically; aedeagus almost straight (Hou and Chen 2010: figs 9–10)	***B. hainanensis***
22	Genital styles forked apically	**23**
–	Genital styles not forked apically	**25**
23	Frons longer at middle line than wide at widest part, about 2.0: 1; basocaudal portion of genital styles in profile produced at a right angle (Yang and Yang 1986: figs 22B, 22H)	***B. membranacea***
–	Frons longer at middle line than wide at widest part, about 2.5: 1; basocaudal portion of genital styles in profile not produced at a right angle	**24**
24	Median portion of genital styles granulate (Huang et al. 1979: figs 8–11)	***B. furca***
–	Median portion of genital styles not granulate (Aschi 1983: fig. 4)	***B. lynchi***
25	Ventral margin of anal segment incised medially; genital styles short, lamellate (Huang et al. 1979: fig. 20)	***B. lacticolorata***
–	Ventral margin of anal segment not incised medially; genital styles slender	**26**
26	Apex of vertex obviously broadened, frons widest at base; apex of genital styles without small teeth; aedeagus short and stout (Huang et al. 1979: fig. 17)	***B. similis***
–	Apex of vertex not broadened, frons widest at apex; apex of genital styles with several small teeth; aedeagus relatively long (Huang et al. 1979: figs 13–15)	***B. citricolorata***

### Bambusiphaga yingjiangensis sp. n.

Taxon classificationAnimaliaHemipteraDelphacidae

http://zoobank.org/416EAEAC-A8BF-4D2F-975E-09CE63A54E0E

Figs 1–9 , 10–21

#### Type material.

Holotype: ♂, **China**: Yunnan Province, Yingjiang County (97°56'E, 24°41'N), on bamboo, 17 Aug. 2015, X.-S. Chen and L. Yang; paratypes, 5♂♂, 23♀♀, same data as holotype.

#### Etymology.

This new species is named after the type locality, Yingjiang, Yunnan Province in China.

#### Measurements.

Body length (from apex of vertex to tip of forewings): male 3.2–3.4 mm (N = 6); female 3.6–3.9 mm (N = 23); forewings length: male 2.5–2.7 mm (N = 6); female 3.2–3.5 mm (N = 23).

#### Diagnosis.

The salient features of the new species include the following: pronotum and mesonotum with blackish brown markings (Figs 3–4); forewings with a large irregular pale brown stripe along transverse vein hence bending along posterior margin to apex (Fig. 8); aedeagus with phallobase, apical 1/4 with three branches (Figs 17–18); genital styles with apical forked (Figs 15–16).

**Figures 1–9.**
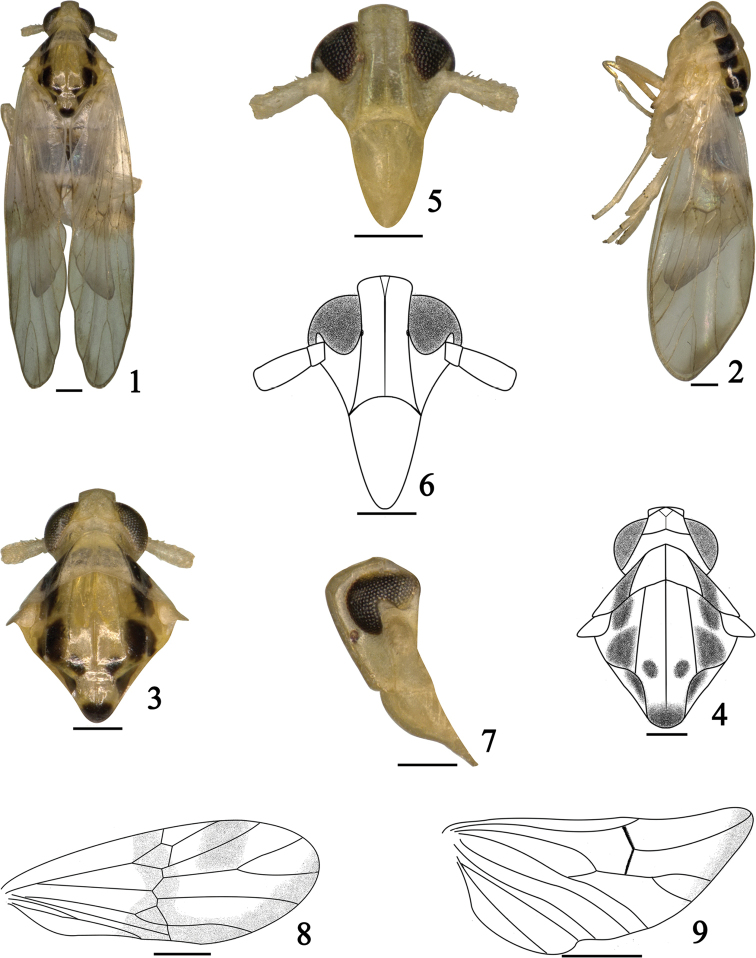
*Bambusiphaga yingjiangensis* sp. n. **1** Male habitus, dorsal view **2** Same, lateral view **3–4** Head and thorax, dorsal view **5–6** Face **7** Frons and clypeus, lateral view **8** Forewing **9** Hindwing. Scale bars **1–7** 0.2 mm; **8–9** 0.5 mm.

#### Description.

*Coloration*. General color light yellow with dark brown markings (Figs 1–2). Vertex, frons, genae, clypeus and antennae light yellow (Figs 1–7). Eyes reddish brown, ocelli red (Figs 5, 7). Pronotum (Figs 3–4) light yellow to yellowish white, outside of each lateral carina with a large dark brown marking. Mesonotum (Figs 3–4) light yellow, outside of each lateral carina with two large dark brown markings, middle area with two small bilateral dark brown markings at apical 1/3, the scutellum with apex dark brown. Forewings (Fig. 8) hyaline, with a large irregular pale brown stripe along transverse vein hence bending along posterior margin to apex, another large pale brown transverse marking from vein Rs+M_1_ to apex of vein Sc_2_. Hindwings (Fig. 9) with a pale brown longitudinal stripe along apical margin.

*Head and thorax*. Vertex with anterior margin broadly rounded, lateral and submedian carinae distinct, ratio width at base to width at apex 1.4, ratio of length to width at base 0.5 (Figs 3–4). Frons with ratio of length at midline to width at widest part 2.2, widest at apex, median carina forked at base (Figs 5–6). Base of postclypeus as wide apex of frons (Figs 5–6). Antennae with basal segment long equal to wide, shorter than second segment (0.4: 1), two segments together reaching to frontoclypeal suture (Figs 5–6). Pronotum with ratio length in midline to length of vertex 1.7 (Figs 3–4). Mesonotum 2.5 times as long as vertex and pronotum combined in middle line (Figs 3–4). Forewings (Fig. 8) longer in middle line than broad at widest part (2.6: 1), apical margin rounded.

**Figures 10–21.**
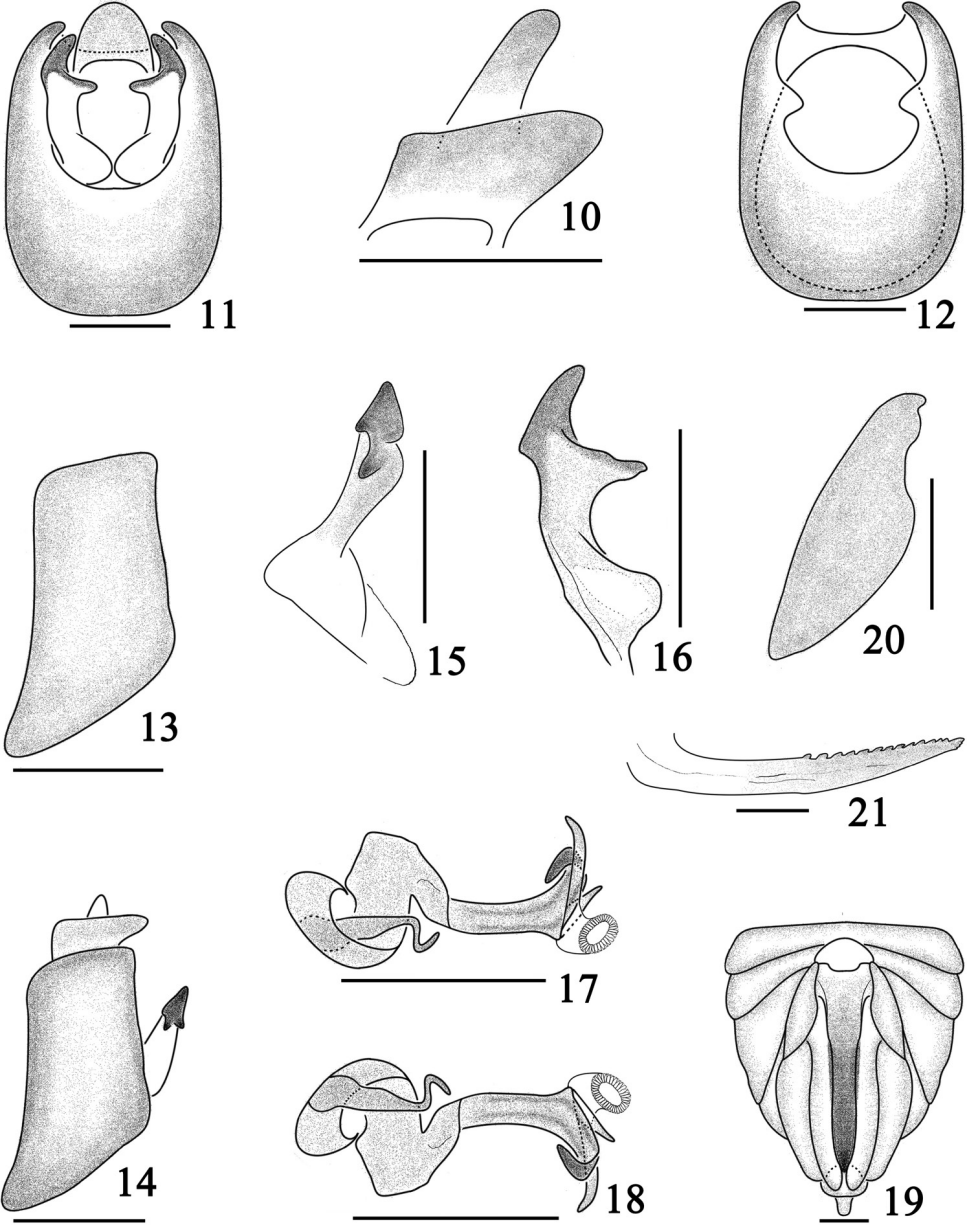
*Bambusiphaga yingjiangensis* sp. n. **10** Anal segment, lateral view **11** Male genitalia, posterior view **12** Pygofer, posterior view **13** The same, lateral view **14** Male genitalia, lateral view **15** Genital style, lateral view **16** Same, posterior view **17** Aedeagus, right lateral view **18** Same, left lateral view **19** Female genitalia, posterior view **20** Gonocoxa VIII, posterior view **21** Gonapophysis IX. Scale bars 0.2 mm.

*Male genitalia*. Pygofer (Figs 12–13) without medioventral process, opening longer than wide in posterior view (Fig. 12), dorsal margin shorter than ventral margin in lateral view (Fig. 13). Aedeagus (Figs 17–18) with phallobase process small and simple, arising from base of aedeagus, with basal 1/2 thick, apical 1/2 thin, S-shaped; phallus complex, apical 1/4 with three branches—the left one curved, directed basad, the middle one small and straight, and the right one, longest slightly curved and directed ventrad; gonopore located at apical 1/4 of phallus, node-like. Genital styles (Figs 15–16) long, with two processes forked at apical 1/3 (Fig. 16), with apex in profile triangular, a large tooth-like located at middle of subapex, directed basad (Fig. 15). Anal segment (Figs 10–11) short, ring-like, without processes, ventral margin convex medially in posterior view (Fig. 11).

*Female genitalia*. Female pygofer (Fig. 19) with gonocoxa VIII moderately large. Ovipositor (Fig. 19) overpassing the pygofer. Gonangulum large, apex blunt, connected gonocoxa VIII. Gonapophyses IX (Fig. 21) curved basally, straight and narrowing apically, dorsal margin with apical 1/2 serrated, ventral margin with three small teeth near the tip.

#### Host plant.

Bamboo.

#### Distribution.

Southwest China (Yunnan) (Fig. 40).

#### Remarks.

This new species resembles *B. nigropunctata* Huang & Ding, 1979, but differs from the latter by: lateral areas of pronotum and mesonotum with several dark brown markings (without dark brown marking in *nigropunctata*); genital styles forked apically (genital styles not forked apically in *nigropunctata*); aedeagus with three branches subapically (aedeagus with two branches apically in *nigropunctata*).

This new species is also similar to *B. taiwanensis* (Muir, 1917) and can be distinguished by: lateral areas of pronotum and mesonotum with several dark brown markings (without dark brown marking in *taiwanensis*); genital styles forked apically (genital styles not forked apically in *taiwanensis*); aedeagus with three branches without tooth (aedeagus with several small teeth on dorsal and lateral sides of the main branch in *taiwanensis*).

Based on the characters of male genitalia, this species should belong to the *nigropunctata* group.

### Bambusiphaga ventroprocessa sp. n.

Taxon classificationAnimaliaHemipteraDelphacidae

http://zoobank.org/3AC6766B-DD2C-4242-9CD9-0EA1702D180D

Figs 22–30 , 31–39

#### Type material.

Holotype: ♂, **China**: Hainan Province, Lingshui County (110°01'E, 18°30'N), on bamboo, 16 Apr. 2017, H.-X. Li; paratypes, 3♂♂, 10♀♀, same data as holotype.

#### Etymology.

The specific name is a combination of the Latin word *venter* (truncated, with o- connecting vowel), meaning belly, ventral; and the Latin word *processus*, meant in the modern biological sense of a projection or appendage, truncated with the feminine termination -*a*.

#### Measurements.

Body length (from apex of vertex to tip of forewings): male 2.4–2.6 mm (N = 4); female 2.4–2.7 mm (N = 10); forewings length: male 2.0–2.2 mm (N = 4); female 2.0–2.3 mm (N = 10).

#### Diagnosis.

The salient features of the new species include the following: forewings with two large black markings at base (Fig. 29); pygofer with mediovental process large and inversed (Fig. 32); aedeagus with numerous inversed spines at apical 1/2 (Fig. 36).

**Figures 22–30.**
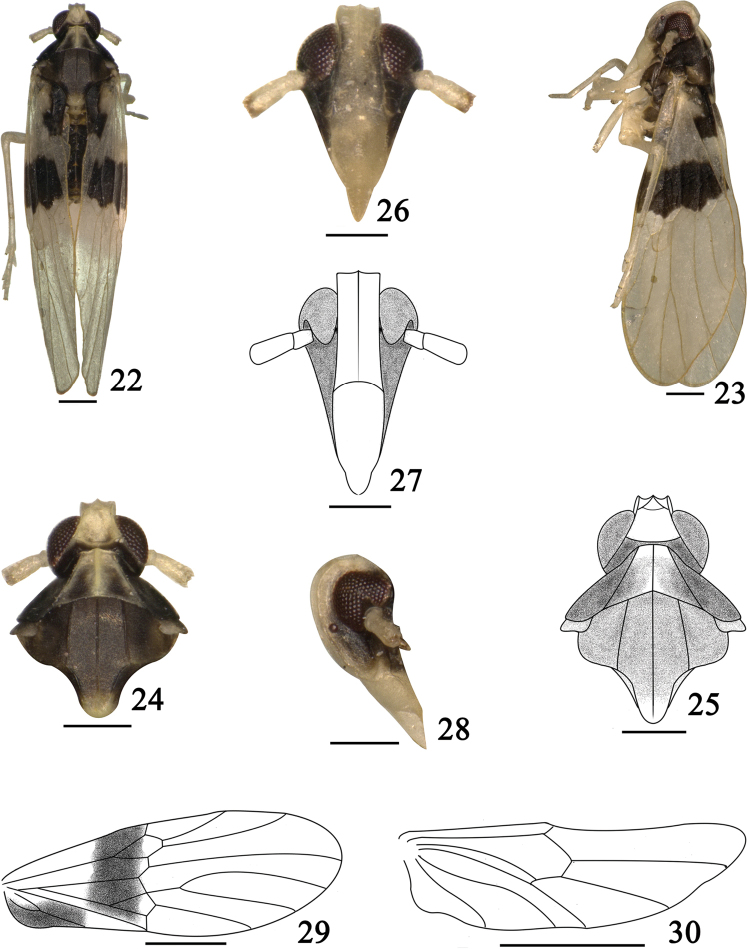
*Bambusiphaga ventroprocessa* sp. n. **22** Male habitus, dorsal view **23** Same, lateral view **24–25** Head and thorax, dorsal view **26–27** Frons and clypeus **28** Same, lateral view **29** Forewing **30** Hindwing. Scale bars **22–28** 0.2 mm; **29–30** 0.5 mm.

#### Description.

*Coloration*. General color yellowish white to black (Figs 22–30). Vertex, frons, clypeus, antennae and legs yellowish white. Genae black brown. Eyes and ocelli brownish red (Figs 26, 28). Pronotum (Figs 24–25) black, disc with anterior 1/3 between lateral carinae and median carina yellowish white. Mesonotum (Figs 24–25) blackish brown, apex of scutellum yellowish white. Forewings (Fig. 29) with two large dark brown markings at basal area.

*Head and thorax*. Vertex (Figs 24–25) with anterior margin angled convex medially, Y-shaped carina with stalk absent, ratio of length to width at base 0.9, ratio width at base to width at apex 1.4. Frons (Figs 26–27) with ratio of length in middle line to width at widest 2.6, widest at apex, median carina simple and obscure apically. Clypeus (Figs 26–27) with width at base as same as frons at apex. Antennae (Figs 26–27) with basal segment subequal to broad, shorter than second segment (1.0: 3.0), reaching to frontoclypeal suture. Pronotum (Figs 24–25) with carinae distinct, lateral carinae attaining hind margin, length in midline as long as vertex. Mesonotum (Figs 24–25) with lateral carinae straight, subparallel, attaining hind margin, median carina obscured apically, ratio length to pronotum and vertex combined in middle line 1.3. Forewings (Fig. 29) with radio of length in middle line to width at widest part 2.5, apical margin rounded. Hindwings (Fig. 30) elongate.

**Figures 31–39.**
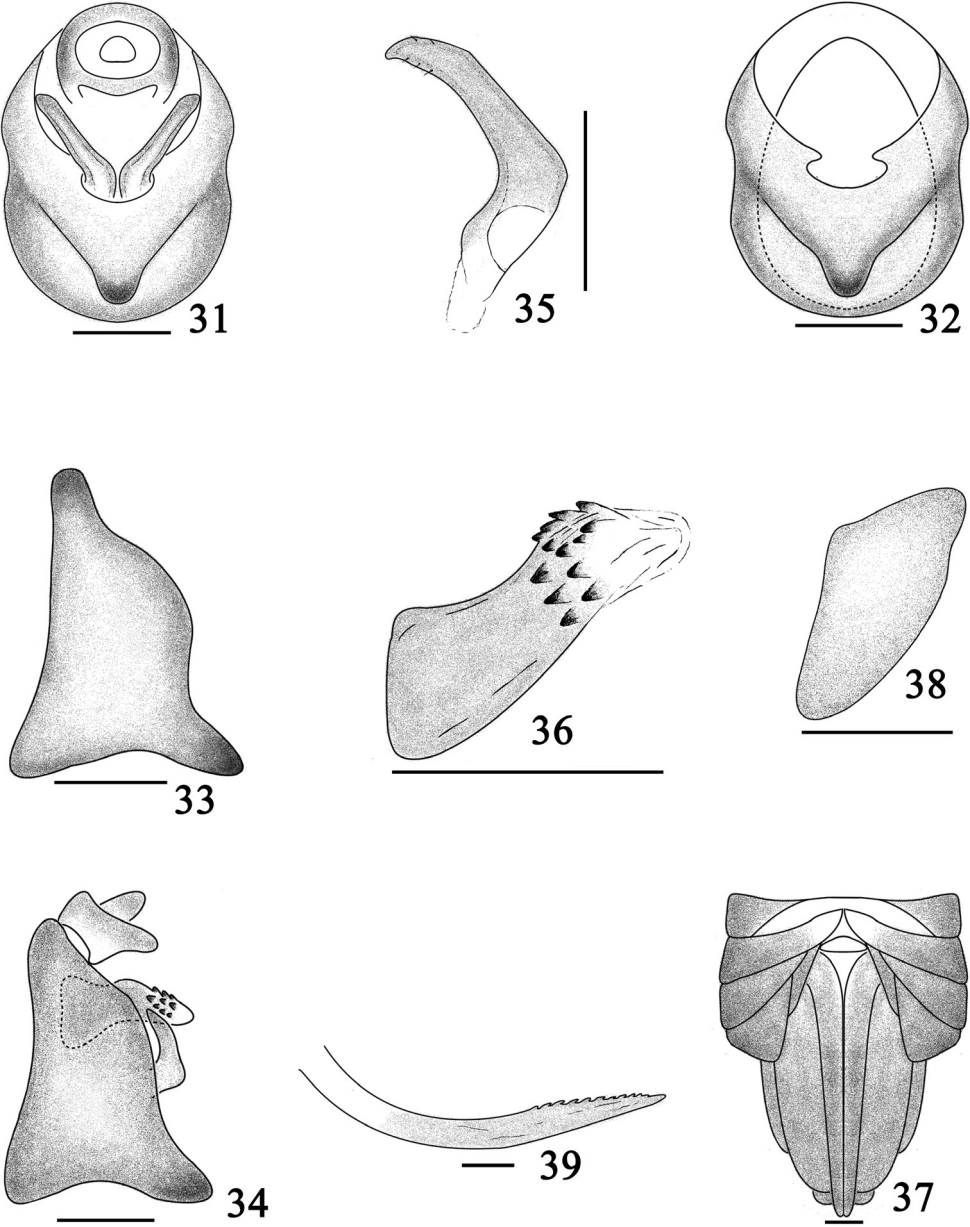
*Bambusiphaga ventroprocessa* sp. n. **31** Male genitalia, posterior view **32** Pygofer, posterior view **33** Same, lateral view **34** Male genitalia, lateral view **35** Genital style, posterior view **36** Aedeagus **37** Female genitalia, posterior view **38** Gonocoxa VIII, posterior view **39** Gonapophysis IX. Scale bars 0.1 mm.

*Male genitalia*. Pygofer in posterior view (Fig. 32) with medioventral process large and inversed, opening longer than wide, lateral margins sinuate; in lateral view (Fig. 33) dorsal margin shorter than ventral margin distinctly, posterior margin concave. Aedeagus (Fig. 36) stout, tubular, apical 1/2 with numerous inversed spines. Genital styles (Fig. 35) moderately long, tapering apically. Anal segment (Fig. 31) short, ring-like, ventral margin without process.

*Female genitalia*. Female pygofer (Fig. 37) with gonocoxa VIII moderately large. Ovipositor (Fig. 37) overpassing apical margin of pygofer distinctly. Gonangulum with apical margin blunt, connected gonocoxa VIII. Gonapophyses IX (Fig. 39) long and large, curved and directed basad, apex sharp, dorsal margin with apical 1/2 serrated.

#### Host plant.

Bamboo.

#### Distribution.

**Figure 40.**
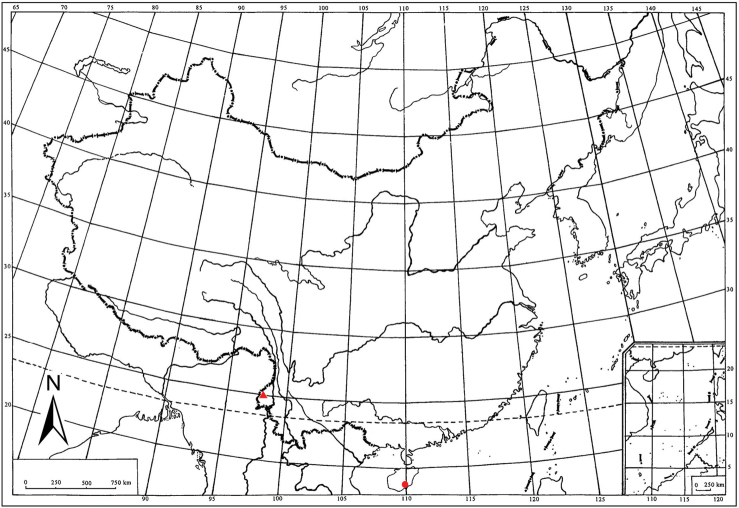
Geographic distributions of two new *Bambusiphaga* species in China: *B. yingjiangensis* sp. n. (▲); *B. ventroprocessa* sp. n. (●).

South China (Hainan) (Fig. 40).

**Figure 41.**
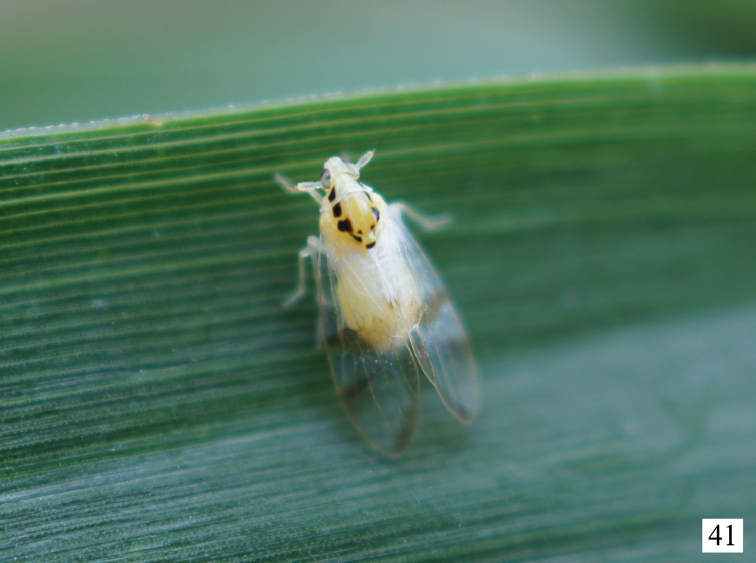
Adult of *Bambusiphaga yingjiangensis* sp. n. resting on leaf of bamboo. Photograph by X.-S. Chen.

**Figures 42–43.**
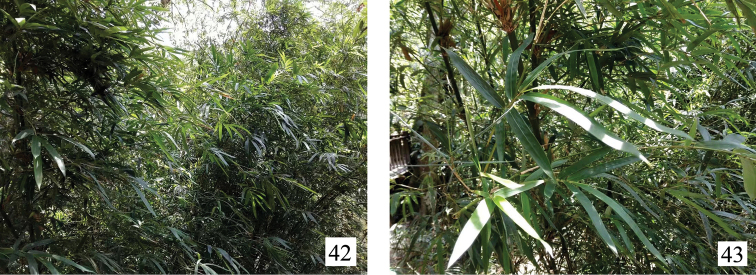
Host plant of *Bambusiphaga ventroprocessa* sp. n. **42** View of the area where the specimens of *B. ventroprocessa* sp. n. were captured, in Lingshui (Hainan, China). **43** View of the plant. Photograph by H.-X. Li.

#### Remarks.

This species is similar to *B. kunmingensis* Yang & Chen, 2011, but can be distinguished by the basal area of forewing with two dark brown markings (forewing with basal 1/3 full dark brown in *kunmingensis*); the mediovental process of pygofer large (without mediovental process in *kunmingensis*); the aedeagus without phallobase (phallobase arising from base of aedeagus, as long as aedeagus in *kunmingensis*).

This new species is also similar to *B. basifusca* Hou & Chen, 2010, but can be distinguished by the ventral margin of anal segment without process (ventral margin of anal segment with a long process in *basifusca*); the ventral margin of pygofer with a medioventral process (ventral margin of pygofer with three medioventral processes in *basifusca*); and the aedeagus without phallobase (aedeagus with phallobase in *basifusca*).

Based on the characters of male genitalia, this species should belong to the *kunmingensis* group.

## Supplementary Material

XML Treatment for Bambusiphaga

XML Treatment for Bambusiphaga yingjiangensis

XML Treatment for Bambusiphaga ventroprocessa
